# PID Controller Design for FES Applied to Ankle Muscles in Neuroprosthesis for Standing Balance

**DOI:** 10.3389/fnins.2017.00347

**Published:** 2017-06-20

**Authors:** Hossein Rouhani, Michael Same, Kei Masani, Ya Qi Li, Milos R. Popovic

**Affiliations:** ^1^Department of Mechanical Engineering, University of AlbertaEdmonton, AB, Canada; ^2^Rehabilitation Engineering Laboratory, Lyndhurst Centre, Toronto Rehabilitation Institute, University Health NetworkToronto, ON, Canada; ^3^Rehabilitation Engineering Laboratory, Institute of Biomaterials and Biomedical Engineering, University of TorontoToronto, ON, Canada

**Keywords:** functional electrical stimulation, balance control, neuroprosthesis, inverted pendulum, neuromuscular modeling

## Abstract

Closed-loop controlled functional electrical stimulation (FES) applied to the lower limb muscles can be used as a neuroprosthesis for standing balance in neurologically impaired individuals. The objective of this study was to propose a methodology for designing a proportional-integral-derivative (PID) controller for FES applied to the ankle muscles toward maintaining standing balance for several minutes and in the presence of perturbations. First, a model of the physiological control strategy for standing balance was developed. Second, the parameters of a PID controller that mimicked the physiological balance control strategy were determined to stabilize the human body when modeled as an inverted pendulum. Third, this PID controller was implemented using a custom-made Inverted Pendulum Standing Apparatus that eliminated the effect of visual and vestibular sensory information on voluntary balance control. Using this setup, the individual-specific FES controllers were tested in able-bodied individuals and compared with disrupted voluntary control conditions in four experimental paradigms: (i) quiet-standing; (ii) sudden change of targeted pendulum angle (step response); (iii) balance perturbations that simulate arm movements; and (iv) sudden change of targeted angle of a pendulum with individual-specific body-weight (step response). In paradigms (i) to (iii), a standard 39.5-kg pendulum was used, and 12 subjects were involved. In paradigm (iv) 9 subjects were involved. Across the different experimental paradigms and subjects, the FES-controlled and disrupted voluntarily-controlled pendulum angle showed root mean square errors of <1.2 and 2.3 deg, respectively. The root mean square error (all paradigms), rise time, settle time, and overshoot [paradigms (ii) and (iv)] in FES-controlled balance were significantly smaller or tended to be smaller than those observed with voluntarily-controlled balance, implying improved steady-state and transient responses of FES-controlled balance. At the same time, the FES-controlled balance required similar torque levels (no significant difference) as voluntarily-controlled balance. The implemented PID parameters were to some extent consistent among subjects for standard weight conditions and did not require prolonged individual-specific tuning. The proposed methodology can be used to design FES controllers for closed-loop controlled neuroprostheses for standing balance. Further investigation of the clinical implementation of this approach for neurologically impaired individuals is needed.

## Introduction

In various neurological conditions, including spinal cord injury, stroke, and traumatic brain injury, a diminished ability to maintain balance during stance is common. Facilitating a stable stance in these populations could offer important benefits, including increased independence and quality of life, as well as decreased risk of secondary complications, such as osteoporosis, urinary tract infections, spasticity, pressure ulcers, and cardiovascular disease (Harkema et al., 2008; Vette et al., 2009; Triolo et al., 2012).

By applying functional electrical stimulation (FES) to contract lower limb muscles (Popović, 2014), multiple groups have attempted to facilitate or improve the standing ability of these clinical populations. Initial attempts at developing such a technology, termed standing neuroprosthesis, utilized open-loop control strategies in which various muscles were stimulated through either transcutaneous or implanted electrodes (Forrest et al., 2007; Fisher et al., 2008). However, open-loop FES systems contract the muscles continuously, resulting in rapid muscle fatigue, an inherent limitation to the degree of achieved stabilization. As a result, affected individuals need to use their upper limbs for support, diminishing their ability to perform the activities of daily living while standing. In order to improve stability and minimize muscle fatigue, a closed-loop feedback controller is required, which turns off or down the stimulation intensity when it is not required based on fluctuations in balance. A number of FES closed-loop control strategies have been investigated, predominantly focusing on plantarflexion/dorsiflexion control of the ankle joints during stance. These have included linear quadratic Gaussian (Hunt et al., 1997; Munih et al., 1997), pole placement design (Hunt et al., 2001; Gollee et al., 2004), H-infinity (Holderbaum et al., 2004), artificial neural network, and sliding mode (Kobravi and Erfanian, 2012) control techniques. However, these control techniques suffer from limitations, including the need for voluntary trunk control, the lack of a strong physiological basis and/or high computational complexity limiting the operating frequency of thecontroller.

The ankle joint plays a major role in standing balance in the sagittal plane, and thus controlling its muscles is the first step in the design and implementation of neuroprosthesis for standing balance. Nevertheless, the clinical implementation of such a neuroprosthesis requires consideration of the balance in the frontal plane as well as closed-loop controlled FES applied to multiple joints of the body. Simulations have demonstrated the feasibility of FES-controlled standing balance using multi-segment models of the body (Jaeger, 1986). Kim et al. have found the optimal choice of lower limb joint angles that should be controlled by FES application to maintain 3D standing balance (Kim et al., 2007). Abbas and Chizeck designed and implemented an FES controller for hip angle control in the frontal plane using percutaneous intramuscular electrodes (Abbas and Chizeck, 1991). An open-loop controlled surgically implanted 8-channel neuroprosthesis for standing has been developed, and clinical studies have shown significant improvement in the quality of life of individuals with spinal cord injury when they used it (Rohde et al., 2012; Triolo et al., 2012). Nataraj et al. showed the efficiency of a comprehensive 3D multi-joint closed-loop control of an implanted FES system and the feasibility of its clinical implementation through simulation (Nataraj et al., 2010, 2012, 2013, 2016). They proposed a method for tuning FES controller parameters for individuals with spinal cord injuries and showed that this system could minimize the loading of the upper limbs needed for standing balance against disturbances. However, a recent review showed that the clinical implementation of the currently available implanted neuroprostheses is limited. As such, function restoration of lower limbs using commercially available surface stimulators and clinical implementation of such a closed-loop controlled FES system for standing is still needed (Ho et al., 2014).

At the same time, previous studies have suggested that the central nervous system (CNS) likely utilizes a feedback mechanism based on changes in both center of mass (COM) displacement and velocity to modulate standing balance (Masani, 2003; Masani et al., 2006; Welch and Ting, 2009; Vette et al., 2010). In order to mimic the control strategy employed by the CNS to maintain standing balance, the proportional and derivative components of a proportional-derivative (PD) control strategy may be employed to account for COM displacement and velocity, respectively. In a closed-loop FES system, a proportional-integral-derivative (PID) controller model can also minimize the accumulated error in the measurement of COM displacement. Our group has already employed PD/PID controllers to model the neural control component of able-bodied stance (Vette et al., 2007, 2009; Tan, 2008) and have experimentally verified the potential of PD/PID controllers to regulate FES amplitudes applied to the ankle flexors (Same et al., 2013; Same, 2014; Tan et al., 2014). In the present study, we proposed a methodology to determine PID controller parameters and implement a PID controller for FES regulation toward maintaining standing balance for several minutes, and in the presence of balance perturbations and inter-subject variability. In the present study, we hypothesize that a PID controller could mimic the intact physiological control strategy for standing balance, effectively modulate FES amplitude levels applied to the ankle plantarflexors and dorsiflexors, and maintain standing stability for several minutes. This hypothesis was examined both through simulations and in experiments using a custom-made apparatus. This study can be the first step toward clinical implementation of a neuroprosthesis for improving the standing balance of neurologically impaired individuals.

## Materials and methods

All of the data collection as well as the closed-loop control were performed using a custom-made program (LabVIEW 2011, National Instruments, USA). All subsequent analyses and computational simulations were performed using Matlab and Simulink (Mathworks, USA).

### The inverted pendulum standing apparatus (IPSA)

**Figure 1:**
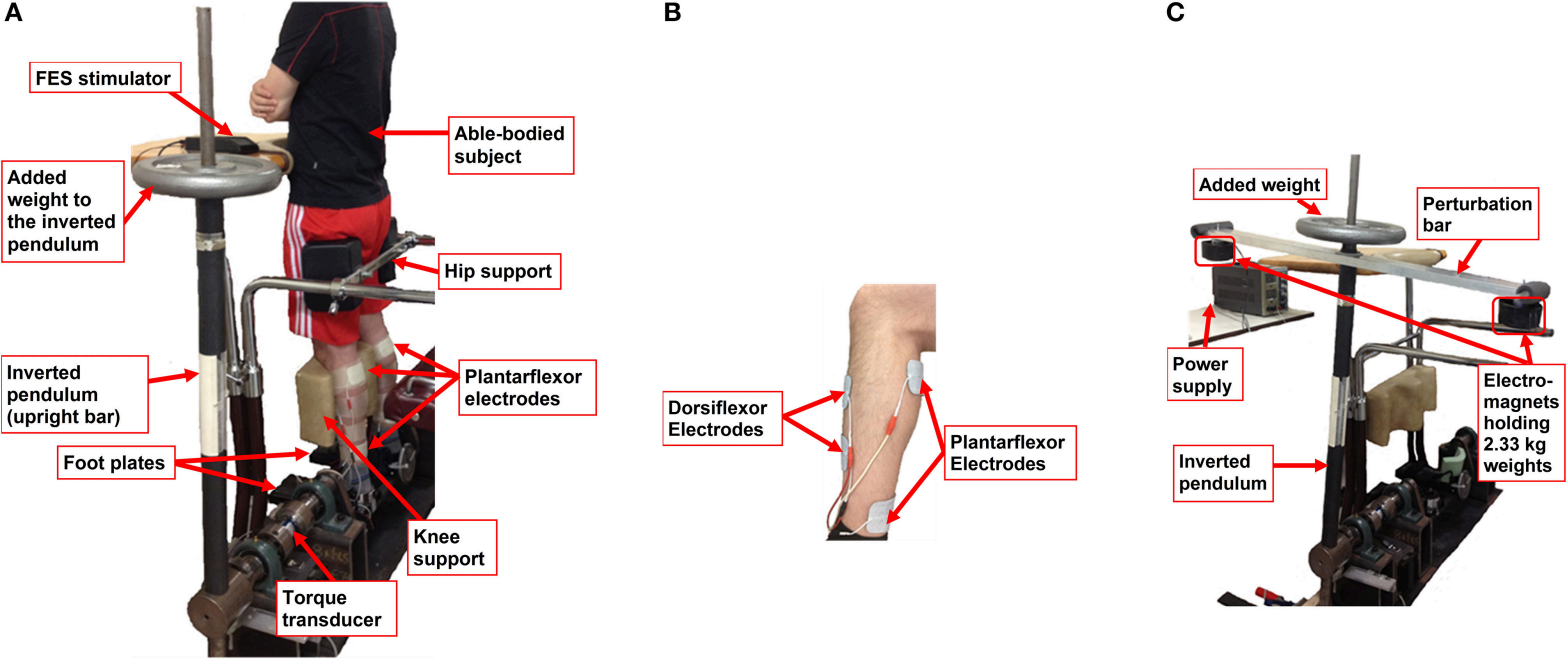
Experimental setup: **(A)** Subject standing in the Inverted Pendulum Standing Apparatus (IPSA) (Same et al., 2013; Tan et al., 2014; Rouhani et al., 2016); **(B)** Electrode placement for bilateral application of FES for standing balance. For plantarflexors, 5 × 9 cm electrodes were applied bilaterally along the midline of the posterior calf. One electrode was placed approximately 2 cm below the popliteal fossa over the gastrocnemius and soleus muscle motor points so as to activate both gastrocnemius heads as well as the soleus muscle. The other electrode was placed around the lower end of the gastrocnemius muscle belly just above the ankle joint. For dorsiflexors, 5 × 5 cm electrodes were applied. One electrode was placed over the motor point of the tibialis anterior, just lateral to the fibula, and the other electrode was placed approximately 8 cm below the anode; **(C)** Experimental setup for testing the FES controller's response to postural balance perturbation. A perturbation bar is added with weights at its two ends. Dropping of each weight at random instants induces perturbation torque applied on the inverted pendulum. This mechanism could simulate the torque induced due to arm motion in the sagittal plane during standing.

The human body flexes during static standing at several joints along its longitudinal axis (Aramaki et al., 2001). However, it is known that an inverted pendulum model can approximate the human body rotating around the ankle joints in the sagittal plane during bipedal static standing (Winter, 1995). This model does not account for movements at the knee and hip joints, or in the medial-lateral direction. However, Gage et al. showed that during quiet standing and in the sagittal plane (i) the ankle angle is highly correlated with the movement of the body COM; (ii) COM displacement for each segment increased linearly with its height relative to the ankle joint; and (iii) the body COM acceleration correlated strongly with the difference between the center of pressure and the COM displacements. These observations all together indicated the validity of this model during quiet standing (Gage et al., 2004). To investigate standing balance based on this model, a human-sized inverted pendulum apparatus, hereafter referred to as Inverted Pendulum Standing Apparatus (IPSA), was created in-house (Tan et al., 2014; Figure 1A). In the IPSA, the subject was supported in an upright, motionless standing position using a mechanical frame which locked the person's knees and hips in an extended posture. In this posture, the subject's feet were placed and fixed via straps on foot plates whose rotational axis was shared with a physical, human-sized inverted pendulum. The closed-loop controlled FES was bilaterally applied on the ankle plantarflexors and dorsiflexors that generated closed-loop controlled ankle torque. In this way, the subject's ankle joints directly controlled the swinging of the inverted pendulum in the sagittal plane. The angle of the inverted pendulum was measured by a laser displacement sensor (LK500, Keyence, Japan; repeatability: 50 μm, equivalent to 0.07 deg for the pendulum angle in our measurement setup) and fed back in real-time. The joint torque exerted by the subject's ankle joints onto the foot plate was measured using a torque transducer (TS11-200 Flange Style Reaction Torque Transducer, Interface, USA; combined error: ± 0.1 %FS; repeatability: ± 0.02 %; sensitivity: 1.0 mV/V-N.m).

This upright, stationary position of the body decreased vestibular and proprioceptive sensory contributions. Visual feedback was also removed by utilizing an eyes closed condition. As such, while sway of the physical inverted pendulum mimicked the subject's body during quiet stance, natural activities in the anti-gravity muscles of the lower limbs could be significantly reduced. We have previously shown that, when a subject stands in this posture, his/her natural muscle activities in anti-gravity muscles are automatically diminished (Masani et al., 2013). Although reducing the natural activity of the anti-gravity muscles does not have clinical pertinence, our experimental setup using IPSA minimized the contributions of natural muscle activities, and thus allowed assessment of the performance of the controller employed in standing neuroprosthesis, even when the subject was an able-bodied individual. As such, difficulties related to testing in patient populations were avoided (Tan et al., 2014).

### Control strategy

**Figure 2:**
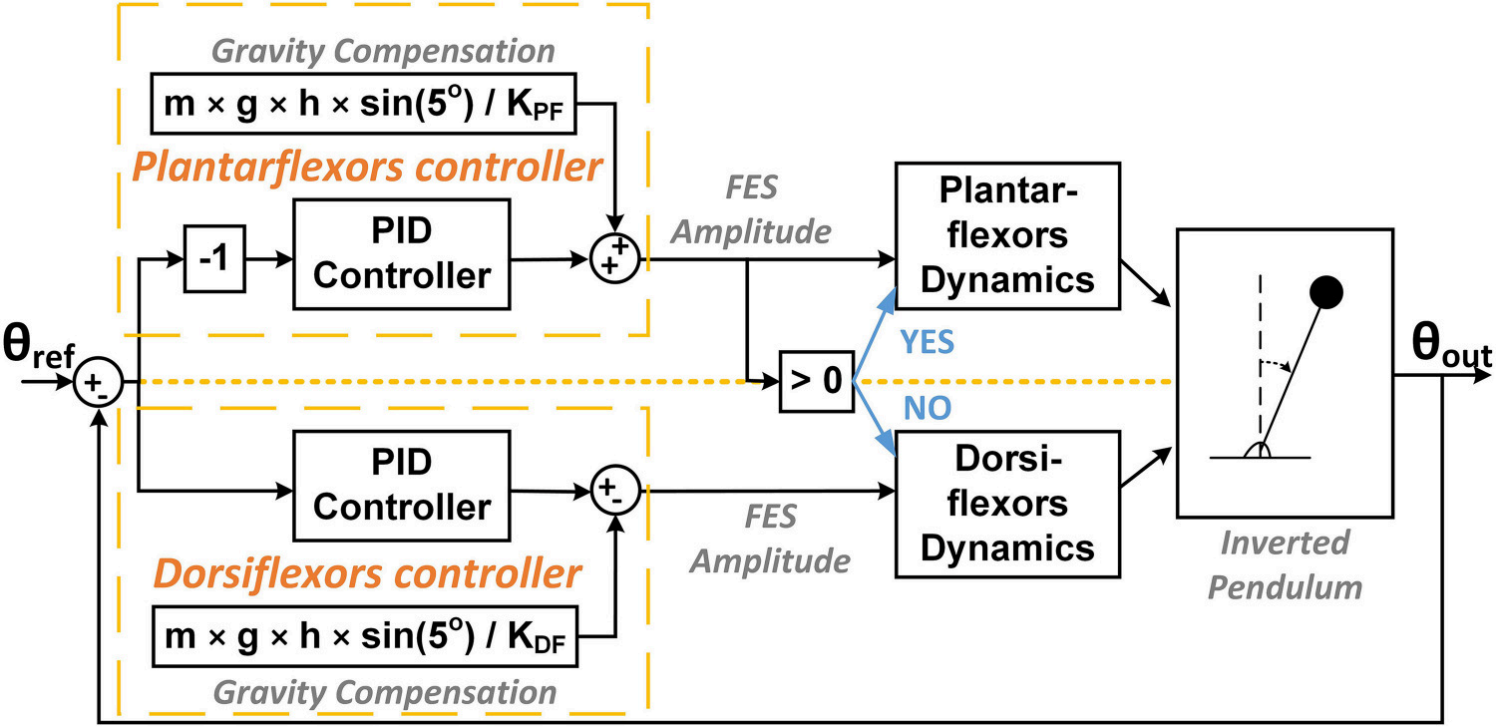
Block diagram depicting the PID plus gravity control strategy.

The PID control strategy employed is summarized in Figure 2. An error signal was obtained by comparing the angle of the inverted pendulum to a reference angle of 5 deg in real-time. This reference angle was selected to simulate a typical standing posture where the whole body COM is located approximately 5 deg anterior to the ankle joint (Masani, 2003). The error signal was fed into individual PID controllers for the plantarflexors and dorsiflexors (Equation 1):

(1) $$C\left(s\right)=K_{P}\left[1+\frac{K_{D}N\cdot s}{s+N}\right]+\frac{K_{I}}{s}$$

where *C(s)* is the transfer function of the PID controller, *K*_*P*_ is the proportional gain, *K*_*I*_ is the integral gain, *K*_*D*_ *is* the derivative gain, and N is the derivative filter gain, incorporated to avoid amplification of high-frequency noise. *K*_*D*_ was defined in proportion to *K*_*P*_ since its ratio to *K*_*P*_ is often a more relevant indicator of its relative contribution (Ang et al., 2005).

The gravity compensator provided the torque required to compensate the gravity toppling torque at the reference angle of the inverted pendulum. For controlling only ankle joint motion while others are locked, the gravity support can be provided by a constant level of FES amplitude applied on ankle flexors. The output of the PID controller gave the FES amplitude that generates the ankle torque required for compensating the deviation of the inverted pendulum angle from the reference angle. The pre-determined dynamic response of muscles, in terms of ankle torque, to FES amplitude was used to convert the FES amplitude into ankle torque (see subsection Target Muscles). The summation of the gravity toppling torque and the PID controlled torque provided the torque required for stabilizing the inverted pendulum at the reference angle.

To mimic the reciprocal activity between the ankle plantarflexors and dorsiflexors, only one muscle was active at any given time. Since the reference angle was 5 deg (simulating the body COM anterior to the ankle joint), the controller mostly required to generate positive torque and thus the plantarflexors were supposed to mostly control the ankle joint torque. The FES applied to the dorsiflexors was activated only when the controller required generating negative torque.

### Participants

Twelve able-bodied (24 ± 5 years old, 64 ± 11 kg, 169 ± 6 cm, five females and seven males) with no known neurological or musculoskeletal disorders participated in this study. Before experimentation, each participant gave written informed consent to participate in the experimental study, in accordance with the Declaration of Helsinki. The protocol was approved by the Rehabilitation Medicine Science Research Ethics Board of the University Health Network.

### Target muscles

Stimulation electrodes were applied bilaterally and on both posterior and anterior sides of the lower leg to facilitate stimulation of the gastrocnemius/soleus muscles (plantarflexors) and tibialis anterior muscles (dorsiflexors), respectively (see Figure 1B).

### Identification of the muscle dynamic response

Before the main experimental trials, we experimentally identified the dynamic response of ankle plantarflexors and dorsiflexors to FES pulse amplitude modulation. For this purpose, the subject was placed in a standing position in IPSA with the footplate fixed horizontally, the knees and hips locked in extension. Note that the subjects' muscle showed no activation in this position as mentioned above. A programmable functional electrical stimulator (Compex Motion II, Compex SA, Switzerland) was used to provide stimulation through surface electrodes. Trains of rectangular, balanced, biphasic and asymmetric FES pulses were applied at fixed frequency (20 Hz), fixed duration (300 μs), and modulating pulse amplitude. FES with sinusoidally varying amplitudes between 20 and 60 mA were applied to the subjects' plantarflexors, and the resulting isometric torque patterns were recorded. The applied sinusoidal frequencies were 0.07, 0.15, 0.3, 0.75, and 1.2 Hz. Each trial lasted at least 10 s and was long enough to record two complete periods. Sinusoidal curves at the same frequencies were fitted to the torque output curves. Since the delay introduced by our experimental setup had stochastic components (see details in (Rouhani et al., 2016)), we separated out a constant delay of 40 ms as the approximate delay introduced by the setup, when applying the sinusoidal fitting. The amplitude gain and phase difference between the input and output sinusoids were obtained for each frequency. The muscle dynamics were then identified using a first-order model (Equation 2):

(2) $$M_{PF}\left(s\right)=\frac{K_{PF}}{1+\alpha_{PF}.s}$$

where *M*_*PF*_*(s)* is the transfer function of the muscle, *K*_*PF*_ is the zero-frequency gain of the muscle model, and α_*PF*_ is the time constant. These first-order models were then incorporated in the closed-loop control system model. A similar procedure was then completed for the dorsiflexors.

### Simulations for PID controller design

**Figure 3:**
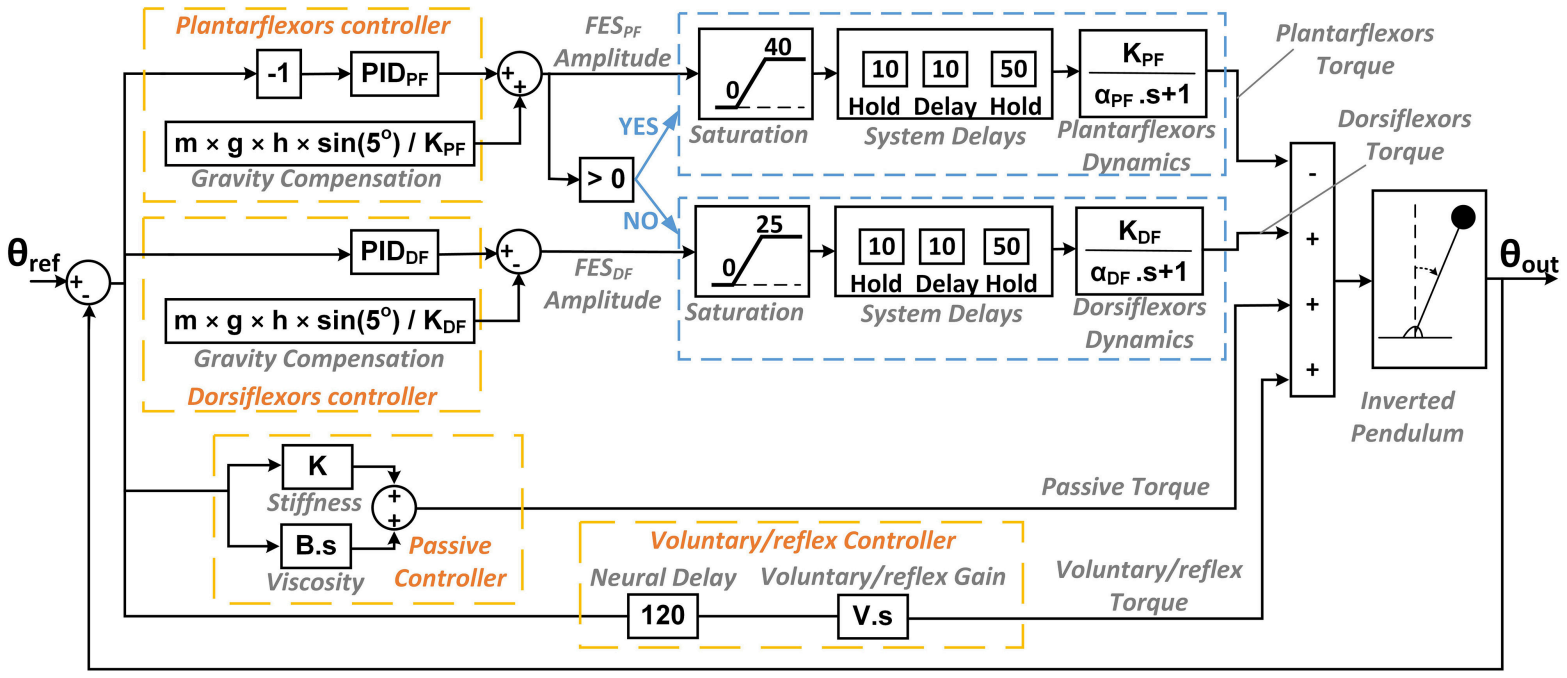
The controller model used in the simulation including the two PID controllers, the gravity compensation component and the mechanism for switching between muscles, and other components added to the controller depicted in Figure 2 in order to model the experimental behavior of the system.

Simulations were performed (using Simulink) to establish controller parameters for use in experiments (Figure 3). PID controllers, gravity compensators, and muscle dynamics components were integrated into the model for plantarflexors and dorsiflexors, separately (See section Participants).

At the output of PID controller plus gravity compensators, saturation blocks were added, limiting the allowable ranges of FES amplitude applied to a subject to between 20 and 60 mA for plantarflexors and between 20 and 45 mA for dorsiflexors. Below the 20 mA minimum levels, the current amplitude dropped to 0 mA. The upper limits of these ranges were chosen so as to minimize subjects' discomfort. Following the saturation blocks and before the muscle models, two Hold blocks were incorporated on either side of a constant delay for each muscle. The first Hold block discretized the signal every 10 ms to reflect the 100 Hz operating frequency of the LabVIEW program. Next, a 10 ms constant delay to account for the internal delay of the FES stimulator. Finally, another 50 ms Hold block was incorporated to model the worst-case scenario, since the frequency of FES pulse trains was 20 Hz and, thus, an additional delay of up to 50 ms could be present.

We also added a “Passive Torque” component to account for the effect of the intrinsic mechanical stiffness of the ankle, which has been previously incorporated in models for standing balance control, in combination with an active component (Peterka, 2000, 2002; Masani et al., 2008; Vette et al., 2010). A stiffness gain and a viscosity gain were included which varied in relation to changes in the inverted pendulum angle and rotational velocity, respectively. The values of these gains were selected based on the literature (Peterka, 2000, 2002; Loram and Lakie, 2002; Masani et al., 2008; Vette et al., 2010; Di Giulio et al., 2013). Following these studies, we used identical gains for all subjects. The lack of measurement of individual-specific passive controller gains for muscles can be a limitation for the clinical implementation of our proposed approach since there may be large inter-subject variability for individuals with neuromuscular impairment.

While the IPSA helps to disrupt neural (voluntary and reflex) control of muscles, it is unreasonable to suggest that voluntary/reflex muscular control would be totally absent. Thus, a voluntary/reflex damping factor was utilized to account for the contribution of voluntary/reflex control in opposition to rotational velocity. The addition of this component makes intuitive sense given that the CNS is likely to automatically activate muscles to some extent in response to rapid movements of the pendulum in order to avoid destabilization, as previously documented (Moore et al., 1988; Santos et al., 2010). In accordance with previous studies, a “Neural Delay” of 120 ms was incorporated to account for the neural-mechanical delay involved in the active control of muscles activity (Moore et al., 1988; Santos et al., 2010).

In order to determine the individual-specific PID controller parameters, we performed simulation trials. In the simulation trials, (i) the initial offset position was modeled using a step function with a negative initial value, (ii) the step response was modeled using a pulse of 8-s duration that shifted the reference angle from 5 to 9 deg, and (iii) external perturbations were modeled as short duration torques applied to the inverted pendulum. Simulations for each subject took into consideration his/her own muscle dynamics. Therefore, the obtained PID controller parameters were individual-specific. An optimization routine based on a two-stage grid search was run to obtain PID controller parameters that minimize the root mean square error (RMSE) between the simulated pendulum angles and reference angle over 40 s. For each subject, first we calculated this angular RMSE value for each combination of the following controller parameters varying at multiple levels on a four-dimensional grid: (i) *K*_*P*_ for plantarflexors; (ii) *K*_*P*_ for dorsiflexors; (iii) *K*_*I*_; and (iv) *K*_*D*_. Note that *K*_*I*_ and *K*_*D*_ were assumed to be identical for both plantarflexors and dorsiflexors. The controller parameters that minimized the angular RMSE for a single step response were determined. Second, the same search procedure was repeated in a smaller four-dimensional window close to these determined parameter values, separately for each of simulation trials (i), (ii), and (iii) described above to fine-tune the optimized controller parameters for each simulation trial and each subject. These optimized PID parameters for plantarflexors and dorsiflexors were recorded for each subject to be used later in the experiments.

### Experimental protocol

After identifying the muscle dynamics and establishing appropriate controller gains through the simulations, the subject was placed in a standing position again in IPSA, with the foot-plate free to move. In the “Standard-weight paradigm,” the total inverted pendulum mass was 39.5 kg, with a center of mass of 0.695 m, and a moment of inertia of 26.7 kg m^2^. These values were chosen for all subjects to avoid rapid muscle fatigue, and, thus, allow for successful testing of all targeted experimental paradigms.

The subjects were instructed to have their eyes closed and arms crossed on the chest during all trials, to attempt to relax, and suppress voluntary control of muscle contractions. The subject wore headphones and listened to whale sounds to limit auditory information and further disrupt sensory input. Then, 10–20 s trials were initiated to test the controller performance and elucidate whether further fine-tuning of controller gains was required. For this purpose, the inverted pendulum was initially inclined at 14 deg, which required bringing it back to the reference position. The tuning procedures were based on a previous study (Li et al., 2006) and our preliminary research. In these short trials, a few variations of PID controller gains were applied in the case of the PID controller performed inadequately (due to potential differences between the modeled and actual closed-loop system). Inadequate controller performance was assessed visually in comparison to other subjects and our preliminary studies in the past, and the adjusted gains were chosen close to the ones suggested by simulation. We used the same optimized controller parameters for the step response with both standard weight and body weight and fine-tuned them for each test paradigm separately.

Once appropriate controller gains were selected, longer trials were performed. Each experimental paradigm was performed both with the FES controller activated (FES condition) and with disrupted voluntary-control and no FES (VOL condition). In the VOL condition, the visual, vestibular, and to some extent the proprioceptive sensory information was suppressed, and the subject was instructed to attempt to balance the inverted pendulum using voluntary control and given auditory cues at the beginning of a trial and for step responses. These trials quantified the subject's ability to balance the inverted pendulum using his/her remaining sensory inputs (somatosensory inputs from the feet and to some extent proprioceptive input from the ankles). Thus, any improvements in performance observed in the FES condition, compared to VOL condition, could be attributed to the PID controller performance. The subject was given approximately 30 s to attempt to balance the inverted pendulum while receiving visual input on the real-time angle, to gain familiarity with the proprioceptive and somatosensory inputs. In order to account for the potential effects of fatigue, the order of the trials was randomized, and the subjects were allowed to rest for at least 3 min in between trials. This timeframe was selected because the majority of recovery has been reported to occur within the first few minutes of rest (Mizrahi et al., 1997; Tepavac and Schwirtlich, 1997).

The experimental paradigms included:

**5-minute quiet-standing (Standard-weight) trials:** This paradigm tested the controller's prolonged ability to maintain the inverted pendulum about the 5 deg reference angle.
**90-second step-response (Standard-weight) trials:** The reference angle changed instantaneously from 5 to 9 deg (upward step) at two instances and each time returned to 5 deg (downward step) after a period of 8–12 s, resulting in a total of four steps. This time period and the initiating instant of each of these two step responses was randomized.
**60-second perturbation (Standard-weight) trials:** Before beginning these trials, a bar was added to the IPSA perpendicular to the inverted pendulum. Electromagnets holding 2.33 kg weights were applied to both ends of the bar (Figure 1C). Then, during a 60-s trial, the power supplied to each electromagnet was removed at different times, resulting in anterior and posterior perturbations of the inverted pendulum, which simulated the perturbation torque due to the subjects moving their arms and lifting objects with their hands. Thus, the ability of the controller to overcome these external perturbations was assessed.
**90-second body weight-matching step-response trials:** The perturbation bar was removed, and extra weights added to the pendulum such that the total mass and COM of the inverted pendulum approximated the subject's mass and COM (“Body-weight paradigm”). Then, step-response trials were performed similar to paradigm (ii).

### Data analysis

For all test paradigms (i)–(iv), the inverted pendulum sway was quantified with RMSE between the pendulum and reference angles, separately for both FES and VOL conditions. The first 10 s of these trials were disregarded to avoid the influence of the initial transient response. Similarly, the RMS of the applied torque and FES current amplitudes applied to plantarflexors and dorsiflexors were calculated in these test paradigms.

In addition, for step response paradigms (ii) and (iv), for both FES and VOL conditions, rise time (to within 10% of the step size of 9–5 = 4 deg), settling time (to within 10% of the step size of 4 deg) and overshoot percentage were also calculated in each step response (total of four steps per trial). “Infinity” was recorded if rise time or settling time criterion was not achieved before the next step occurring at least 8 s later. The median of the four values of rise time, settling time, and overshoot was reported for each trial. A more restrictive definition of settling time was not employed since small fluctuations of body sway in quiet standing is considered natural and, thus, maintaining a precise reference angle was never aimed.

Statistical analyses were performed to test for significant differences between responses in FES and VOL conditions for any of the paradigms examined. For all measures, the Kolmogorov-Smirnov test rejected the null hypothesis that the data came from a normal distribution. Therefore, we employed the non-parametric Wilcoxon Signed Rank test throughout with significance level set at 0.05. Median value of each parameter among all subjects was used to represent the parameter for the group in the Results section.

## Results

Three subjects were not able to tolerate the discomfort due to FES application in the Body-weight paradigm and, thus, only nine subjects participated in test paradigm (iv). Because of technical challenges or subject preference, the duration of the measurement trials for a few other subjects was altered, as reported in Table 1.

**Table 1:** The duration of each test paradigm (in seconds) for individual subjects.

**Subject no**.	**Standard weight**						**Body weight**	
	**Quiet standing**		**Step response**		**Perturbation**		**Step response**	
	**FES**	**VOL**	**FES**	**VOL**	**FES**	**VOL**	**FES**	**VOL**
1	300	300	90	90	60	60	90	90
2	300	300	90	90	60	60	90	90
3	90	90	90	90	90	90	90	90
4	300	300	90	90	90	90	90	90
5	180	200	90	90	90	90	90	90
6	300	300	90	90	60	60	90	90
7	300	300	90	90	60	60	90	90
8	300	300	90	90	60	60	90	90
9	300	300	90	90	60	60	90	90
10	200	200	90	90	60	60	0	0
11	300	300	90	90	60	60	0	0
12	300	300	90	90	0	0	0	0

*FES, FES controlled balance; VOL, disrupted voluntary control of muscle contractions*.

**Figure 4:**
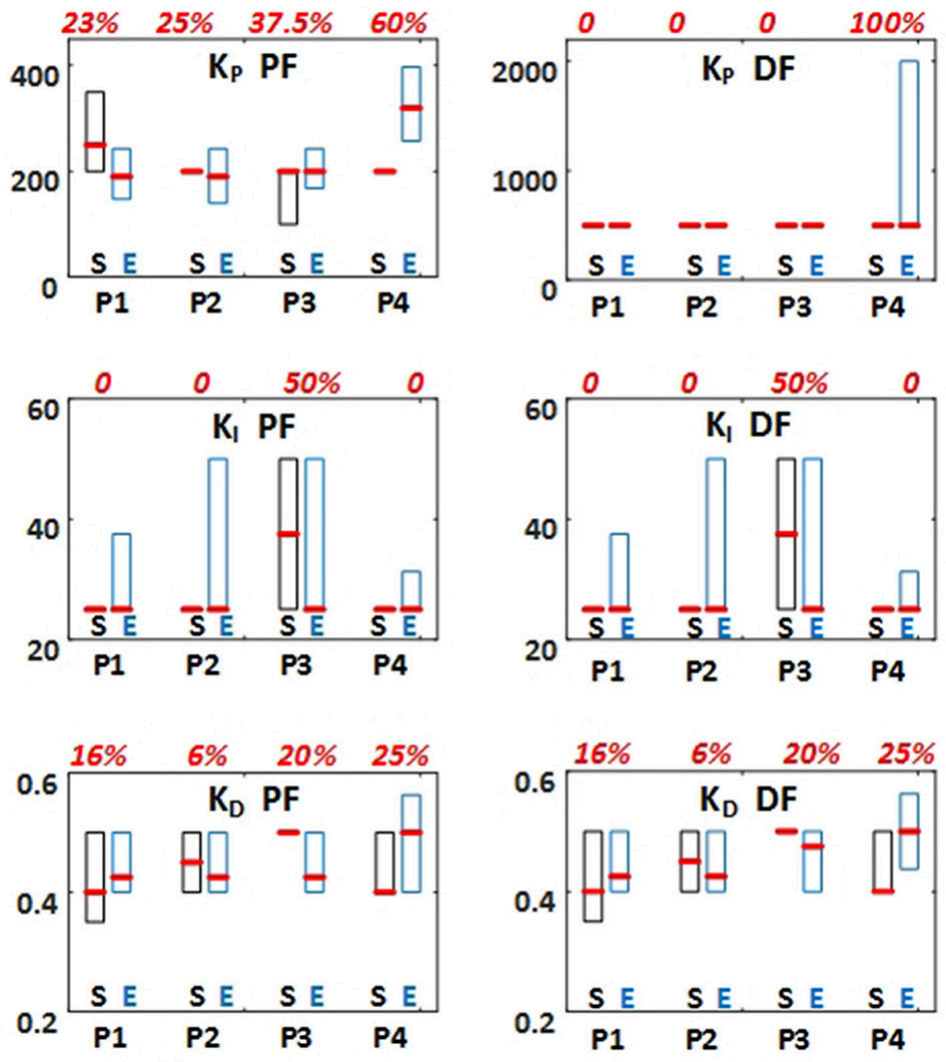
PID controller parameters (K_*P*_, K_*I*_, and K_*D*_) used in experiments for all test paradigms (P1–P4). The values were first suggested through simulations based on the inverted pendulum and muscle dynamics models (S: Simulation). Then, they were tuned within preliminary experiments. Finally, they were applied for the main experimental tests (E: Experiment). The results are presented as box plot indicating 25%, 50%, and 75% percentiles among all subjects. Boxplots related to simulations are shown is black and those related to experiments are shown in blue. The absolute value of the differences between the values obtained through simulation and those implemented experimentally, relative to the values obtained through simulation (in percentage) were calculated. The median of these values among the subjects are presented in red above each two simulation and experimental boxplots.

The PID parameters (*K*_*P*_*, K*_*I*_*, K*_*D*_) obtained through simulations required further individual-specific tuning. This tuning was characterized with the absolute value of the difference between the values obtained through simulation and those implemented experimentally, relative to the values obtained through simulation. *K*_*P*_ for plantarflexors required between 23% and 60% tuning (median values among subjects) across the four test paradigms (Figure 4). *K*_*P*_ for dorsiflexors required 100% tuning only when body weight was applied [paradigm (iv)]. *K*_*I*_ required 50% tuning only when perturbation was applied [paradigm (iii)]. *K*_*D*_ required 6% to 25% tuning across the four test paradigms. Nevertheless, the Wilcoxon Signed Rank test revealed that there was no significant difference between the controller parameters obtained through simulation and those implemented experimentally for all subjects, for all test paradigms. Therefore, no systematic tuning for all subjects was required. In addition, the inter-subject variability of the PID parameters (*K*_*P*_*, K*_*I*_*, K*_*D*_) in Standard-weight paradigms was characterized by the ratio of interquartile range to median, which was <50%, 25%, and 100% for *K*_*P*_*, K*_*D*_, and *K*_*I*_, respectively. This indicates the extent of the similarity in the PID parameters among subjects.

**Figure 5:**
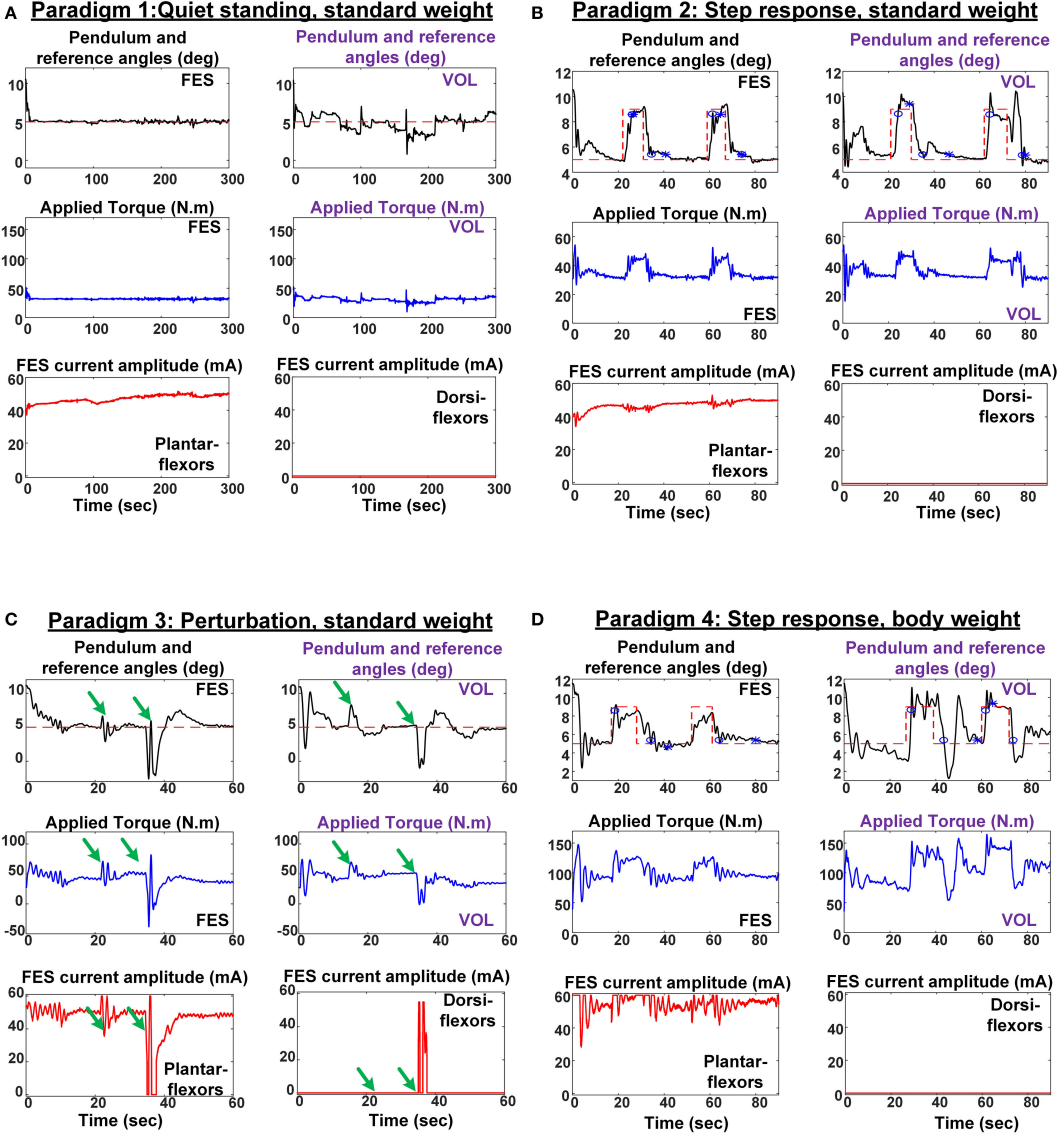
Results for the four experimental paradigms: The inverted pendulum (black) and reference (red dashed) angles and applied torque (blue) are presented for FES control (black titles) and voluntary control (purple titles) conditions. In addition, FES pulse amplitude applied to plantarflexors and dorsiflexors in FES control condition are presented. Results are presented for a representative subject in Test paradigm (i), Quiet standing, Standard weight **(A)**; Test paradigm (ii), Step response, Standard weight **(B)**; Test paradigm (iii), Perturbation response, Standard weight **(C)** (In this figure, the instants of perturbation application are shown by green arrows); Test paradigm (iv), Step response, Body weight **(D)**. In this figure, Rise time (blue circle) and settling time (blue asterisk) are shown for each step in both conditions.

Figure 5 shows the representative examples of the pendulum angle in the FES condition compared to the VOL condition for a representative subject in all test paradigms. Similar ranges of the generated ankle torque were observed between these two conditions. The FES amplitude applied to the plantarflexors was qualitatively larger in Body-weight paradigm [paradigm (iv)] compared to other paradigms. FES on the dorsiflexors was activated rarely and only in paradigm (iii) for this subject.

**Figure 6:**
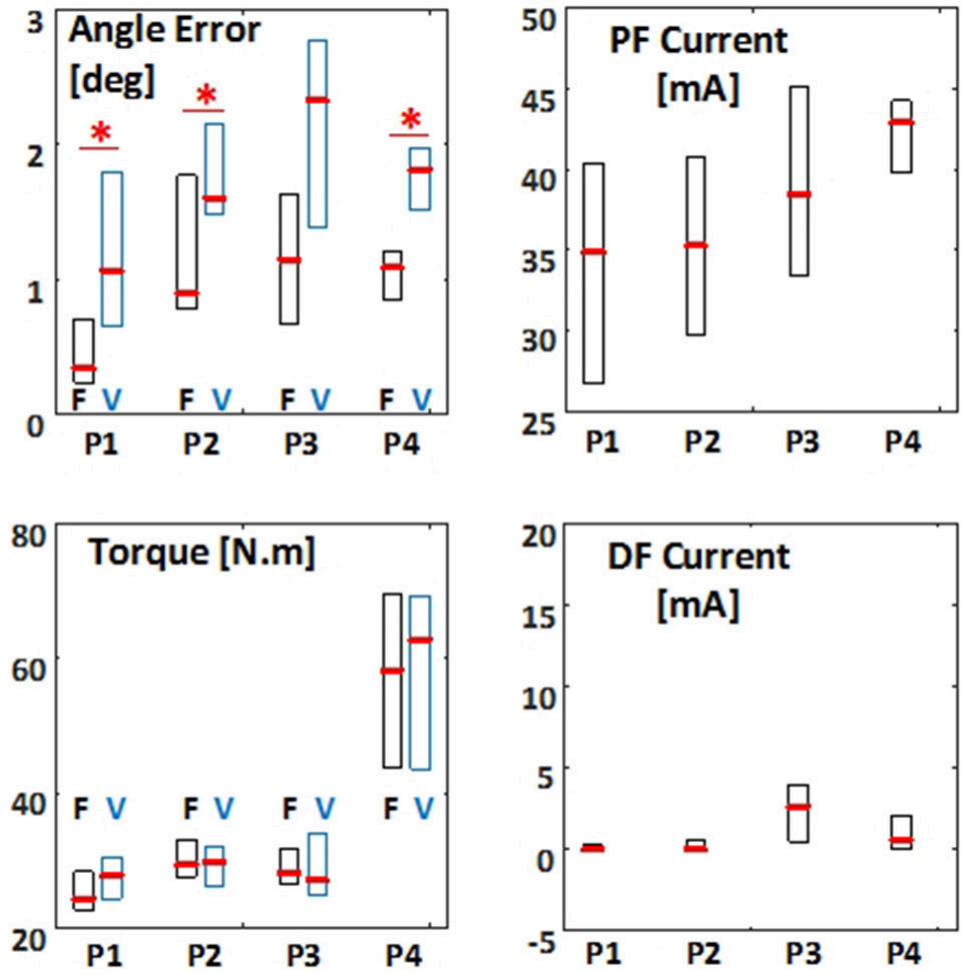
The root mean square (RMS) of the error between the pendulum angle and reference angle, applied torque, and applied FES pulse current amplitude to plantarflexors (PF) and dorsiflexors (DF) for different test paradigms. F indicates FES controlled balance, and V indicates disrupted voluntary control of muscle contractions. The results are presented as boxplot among all subjects, for all test paradigms (indicated with P1–P4). Boxplots related to FES condition are shown is black and those related to VOL condition are shown in blue. Asterisk indicates significant difference (*p* < 0.05) between FES and VOL conditions.

Figure 6 shows the group results of the balance performance for the FES and VOL conditions. In quiet-standing and step-response paradigms [paradigms (i) and (ii)], the RMSE of the inverted pendulum angle had a median of 0.3 deg and 1.1 deg, respectively, among subjects, in FES condition. These RMSE values were significantly smaller [*p* = 0.027 for both paradigms (i) and (ii)] than those obtained in VOL condition (1.1 deg and 1.6 deg). The applied torque by the ankle joint generated in FES condition had RMS values of 24.4 and 29.4 N.m (median) in paradigms (i) and (ii), respectively. These torque values in FES condition were not significantly different with those observed in VOL condition [27.8 and 29.8 N.m in paradigms (i) and (ii), respectively]. The RMS of the FES amplitude applied to the plantarflexors for generating such ankle torques were 34.9 and 35.3 mA, in paradigms (i) and (ii), respectively. Usually, no FES was applied to the dorsiflexors in these two paradigms (median: 0 mA).

The PID controller applied to FES was able to maintain the stability of the pendulum in response to perturbations, in paradigm (iii). In paradigm (iii), the RMSE of the pendulum angle obtained in FES condition (1.1 deg) tended to be smaller than that obtained in VOL condition (2.3 deg), although the difference was not significant (Figure 6). In addition, the ankle torque measured in FES (28.2 N.m) and VOL (27.1 N.m) conditions were not significantly different. The FES amplitudes applied to plantarflexors to generate the abovementioned ankle torque was 38.5 mA. Similar to paradigms (i) and (ii), the applied FES amplitude was tolerable for our subjects. Unlike paradigms (i) and (ii), FES on dorsiflexors needed to be activated in paradigm (iii) (RMS value of the FES amplitude: 2.6 mA).

According to Figure 6, the PID controller applied to FES in Body-weight paradigm [paradigm (iv)] had a similar performance with Standard-weight paradigm [paradigm (ii)]: (1) The RMSE of the pendulum angle obtained in FES condition (1.2 deg) was smaller (*p* = 0.027) compared to VOL condition (1.9 deg); (2) The generated torque in the ankle joint was not significantly different between FES condition (58.2 N.m) and VOL condition (62.9 N.m); (3) The FES amplitude applied to plantarflexors was tolerable for our subjects (42.9 mA) and usually required no FES applied to dorsiflexors (0 mA).

**Figure 7:**
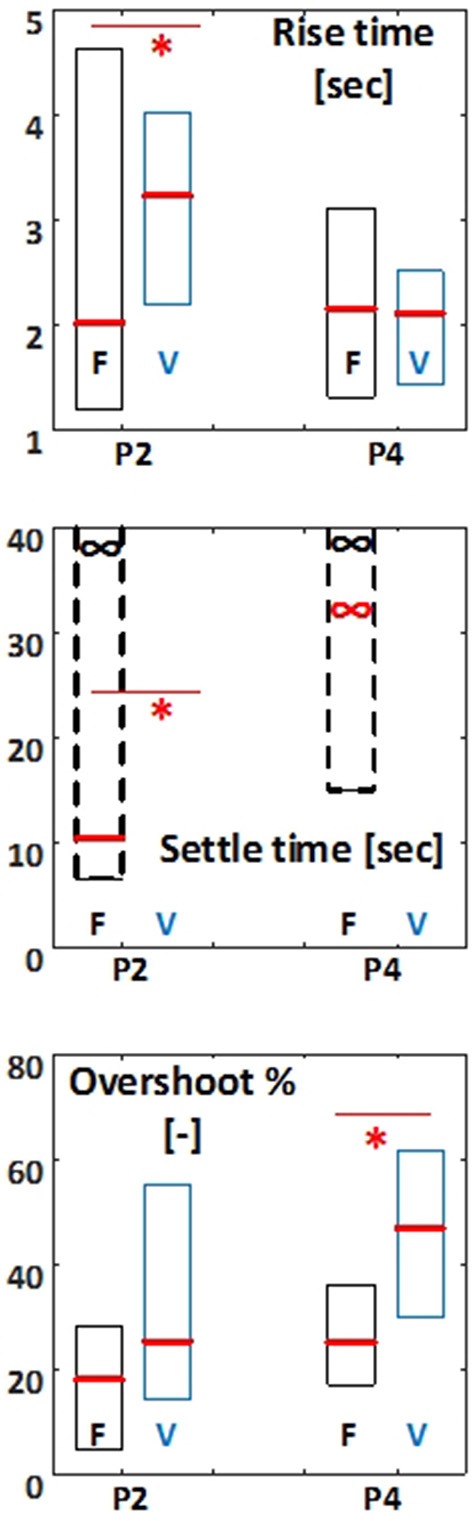
Rise time (s), Settle time (s), and Overshoot (%) for test paradigm 2 (P2: standard weight) and paradigm 4 (P4: body weight). For each test the median parameter for the four steps is reported. F indicates FES controlled balance, and V indicates disrupted voluntary control of muscle contractions. The results are presented as box plot percentiles among all subjects. Boxplots related to FES condition are shown is black and those related to VOL condition are shown in blue. Asterisk indicates significant difference (*p* < 0.05) between FES and VOL conditions. Red and black (∞) signs indicate the 50% and 75% percentiles, respectively, at infinity.

In addition to the steady-state response, the transient response of the PID controller applied to FES showed improvement compared to the disrupted voluntary-control of balance (Figure 7). In test paradigm (ii), the rise time obtained in FES condition (median among all steps and all subjects: 2.0 s) was significantly smaller (*p* = 0.0 034) that that obtained in VOL condition (3.2 s). Median of the settle time among all steps and all subjects obtained in FES condition was 10.4 s. The settle time criterion was not even achieved before the next step in VOL condition for most subjects. Although the overshoot tended to be smaller in FES condition (median: 18.1%) compared to VOL condition (25.2%), this difference was not significant. In paradigm (iv), the difference between rise time in FES (2.2 s) and VOL (2.4 s) conditions was not significant. The settle time criterion was not achieved for most subjects in both FES and VOL conditions. However, the overshoot was significantly smaller (*p* = 0.047) in FES condition (25.2%) compared to VOL condition (46.9%).

## Discussion

This study proposed a methodology for designing PID controllers for closed-loop controlled surface FES applied to ankle plantarflexors and dorsiflexors, as a neuroprosthesis for standing balance. The designed PID controllers were experimentally tested for up to 5 min and in the presence of perturbations and were able to improve standing balance. It was the first time that PID controllers for closed-loop controlled surface FES were successfully implemented on 12 subjects without prolonged individual-specific tuning. Our proposed approach can thus facilitate clinical implementation of neuroprostheses for standing balance without the need for skilful engineers and prolonged tuning of the controller, before each FES trial. Such a device can then be applied in rehabilitation procedures for improving standing balance.

### Controller modeling: PID controller for one-segment inverted pendulum balance

Our proposed FES controller and experimental setup (IPSA) assume a one-segment inverted pendulum model of the body rotating around the ankle joint. Although the knee and hip joints contribute to maintaining standing balance, previous studies using similar devices have shown that the kinematics and dynamics of the able-bodied body during quiet standing are highly correlated with those of an inverted pendulum (Fitzpatrick et al., 1994; Loram and Lakie, 2001, 2002). Whatsmore, the validity of this model during quiet standing in the sagittal plane has been proven (Gage et al., 2004). This verifies the applicability of the inverted pendulum model and its implementation using the IPSA.

Using an inverted pendulum model, previously designed surface FES controllers for standing balance have practically been implemented on up to three subjects and/or for <1 min (Hunt et al., 2001; Gollee et al., 2004; Holderbaum et al., 2004; Mihelj and Munih, 2004; Kobravi and Erfanian, 2012). Consequently, there was still a need for assessing their performance in the long-term and for several subjects before their clinical implementation. Furthermore, previous studies usually suggested the use of a complicated controller for this purpose, which did not necessarily have physiological relevance. Evidences exist that the CNS utilizes feedbacks based on changes in both COM displacement and velocity to modulate standing balance (Masani, 2003; Masani et al., 2006; Welch and Ting, 2009). Therefore, to mimic the physiological control strategy for maintaining standing balance, the FES controller should use such feedback, for example in a PD controller structure. Adding an integral component in our proposed controller may not have specific physiological relevance. Nevertheless, for experimental implementation, we applied a PID controller in the FES controller to minimize the accumulated errors (steady-state offset) due to the measurement errors of COM displacement, mass, COM height and offset reference angle, which may have inaccuracies. In addition, an integral component would reduce the steady-state offset due to model uncertainties, such as muscle behaviors that were not taken into account in our study (e.g., muscle fatigue, reflex, and spasm). For example, in Figure 5, the FES amplitude applied to the plantarflexors showed slight drift over time while the generated torque did not drift visibly. This drift can be because of muscle fatigue and its effect on the muscle response to FES. Notably, these muscle behaviors would be more pronounced in neurologically impaired individuals, and we believe that the integral component of the PID controller contributes to reducing steady-state or accumulated errors over time. The PID controller is known as an easy-to-use and practical controller for many applications that utilizes feedback from both displacement and velocity. However, its comprehensive design and implementation for closed-loop control of FES have been lacking. Preliminary studies of our group on an individual with complete spinal cord injury (ASIA-A) (Tan, 2008) and three able-bodied individuals (Same et al., 2013) showed the potential of the PID controller to regulate FES amplitudes applied to the ankle flexors and to improve standing stability using the IPSA. However, the selection of appropriate controller parameters was not thoroughly investigated. The present study increases the physiological relevance of this previous research by developing a systematic method of selecting controller parameters, using an inclusive model for CNS control strategy and muscle dynamics. This was also the first time that a systematic method for surface FES controller design for standing balance has been verified for several subjects, for up to 5 min, and in a variety of experimental paradigms.

### FES controller vs. voluntary control of muscle contraction

The IPSA can suppress vestibular, visual and some proprioceptive sensory inputs and in fact, cause a disruption to voluntary control. Therefore, instead of impaired balance control in neurologically impaired individuals, eyes-closed voluntary control of balance in able-bodied individuals using IPSA (VOL condition) could be used, for comparison with the FES condition. As such, we would be able to investigate the efficiency of the PID controller for FES when voluntary control of balance is disrupted. Notably, the VOL condition represents the disrupted voluntary control of balance and cannot be used to compare the FES condition with intact voluntary balance control. In this way, potential difficulties related to recruitment, heterogeneity and safety in patient populations were avoided. Figures 5–7 show improved balance for the FES condition over the VOL condition in different test paradigms, in terms of RMSE between the controlled and reference angle, and transient response of the controller (characterized with rise time, settling time, and overshoot). Our observations can indicate the PID controller's potential to compensate for impaired voluntary control in neurologically impaired individuals, although eventually a multi-joint control strategy should be implemented for clinical applications. At the same time, there was no significant difference between the torque applied in FES and in VOL conditions, which indicates that the extent of mechanical effort required by the two controllers is similar (Figure 6). Moreover, the applied FES amplitudes were within a tolerable range for our subjects (Figure 6), and thus this controller can be practical for the application of surface FES electrodes. Despite its simple structure, the PID control strategy was even able to maintain balance in the body weight-matching paradigm and demonstrated efficient performance even in the presence of perturbations. Notably, our subjects had little training with FES, and we expect that future users of this neuroprosthesis would not need extensive FES training before using it.

### PID controller performance

We chose both Standard-weight and Body-weight experimental paradigms to (a) assess the FES controller performance across all subjects for stabilizing the same weight and size of an inverted pendulum to assess the inter-subject variability of the controller performance; and (b) investigate the possibility of maintaining the balance of an individual-specific, body-weight matched inverted pendulum. The long-term (5 min) trial was assessed only once to minimize the influence of muscle fatigue on other experimental paradigms. Nevertheless, our additional 5-min quiet-standing experiments on a few subjects showed this capability in the designed controller.

According to Figure 4, the inter-subject variability of *K*_*P*_ for plantarflexors was due to different muscle strength and response to FES among subjects. We expect the value of *K*_*P*_ to be different for clinical populations, such as individuals with spinal cord injuries. *K*_*D*_ showed relatively small inter-subject variability (<25%). For most subjects and test paradigms, *K*_*I*_ equal to 25 was selected in simulation and experimentally implemented. Because of the rare need for the activation of dorsiflexors, we assumed the same *K*_*D*_ and *K*_*I*_ to those considered for plantarflexors and assumed an identical value of *K*_*P*_ for all subjects in the simulation phase. These controller parameters for the dorsiflexors were later tuned during experiments.

The controller parameters obtained through simulations required individual-specific tuning, often <25% of the values obtained through simulation (Figure 4). This was because of the difference between the actual and modeled systems. In general, *K*_*P*_ for plantarflexors required greater tuning than other parameters. This may be due to the inter-subject variability of passive controller parameters. We were not able to experimentally assess these parameters and thus used the same value based on the literature for all subjects. Test paradigms (iii) and (iv) required more tuning than paradigms (i) and (ii). We believe that in paradigms (iii) and (iv), the neuromuscular system showed greater nonlinearity and system delay and thus our introduced model in Figure 3 was less accurate. Adding nonlinear elements to the muscles and controllers model when a large load or perturbation is applied can improve the accuracy of closed-loop system's model.

Since investigating the best optimization routine was not targeted in our study, our optimization approach was based on a basic grid search method. Although more advanced optimization techniques would be more efficient and faster in determining the optimal parameters, our grid search optimization approach required only a few minutes to determine the controller parameters and our simulations did not show a major change in the controller performance as well as moderate variation of controller parameters.

Our results for RMSE of pendulum angles (<1.2 deg, Figure 6) were smaller than (or at least comparable to) the previous studies that utilized more complicated control strategies (Kobravi and Erfanian, 2012). Note that our employed control strategy was similar to the control strategies employed by the CNS for standing balance in the literature (Peterka, 2000, 2002; Masani, 2003; Masani et al., 2006). In addition to the choice of a physiologically relevant control strategy, our controller model included physiologically relevant time delays, passive control components and a model of voluntary control of muscle contractions. This physiological relevance may justify the choice of PID controller for FES regulation and its efficiency despite its simplicity.

### Limitations and future directions

The successful implementation of the designed PID parameters demonstrated the potential for our suggested methodology to be applied in a wide range of clinical settings as neuroprosthesis. However, it should be noted that this approach was only tested for able-bodied individuals and must be further verified for individuals with different types of neuromuscular impairment. The clinical implementation of our proposed approach for neurologically impaired individuals (e.g., individuals with spinal cord injury) requires further modeling of muscle response to FES, which may be challenging (Nataraj et al., 2012). Some of these challenges associated with this population are the limited muscle force actuation, the nonlinear and less predictable response of muscles to FES, and the impaired muscle reflexes that would be different to those modeled in this study.

Although the ankle joint contributed significantly to maintaining standing balance, the roles played by knee and hip joints as well as upper limbs motion must be further studied to develop the design of a 3D multi-joint FES controller that is required prior to clinical implementation of a neuroprosthesis for standing balance (Vette et al., 2009; Nataraj et al., 2016). In addition, we studied standing balance only in the sagittal plane. A multi-joint FES controller can maintain standing balance in frontal plane as well. Nataraj et al. proposed an approach that applies FES to multiple body muscles to control multiple joints, which makes clinical implementation of the neuroprostheses more realistic. Their FES controller required efforts by upper extremities. They tested this approach through simulation and demonstrated the potential for experimental implementation on individuals with spinal cord injury (Nataraj et al., 2010, 2012, 2013, 2016).

Although we assessed the robustness of the controller against perturbations [test paradigm (iii)], we did not further investigate the PID performance sensitivity to the PID gains (controller parameters) through additional measurements in order to prevent muscle fatigue in subjects. Nevertheless, in our simulations, moderate variation of these gains did not result in a major change in the controller performance, in terms of angular RMSE. Notably, the sensitivity of the controller performance to measurement errors could lead to additional challenges particularly because of the small ranges of angular errors with respect to the reference angle (Nataraj et al., 2010). This was not targeted in our study but should be studied before clinical implementations on individuals with spinal cord injuries.

Our proposed controller for FES was tested for quiet standing with small ranges of standing excursion. It should be further tested for the control of a larger body sway that requires multi-joint closed-loop controller FES application. Moreover, the FES on the dorsiflexors was activated in the presence of perturbation [paradigm (iii)] and rarely in other test paradigms. Therefore, we were not able to assess the sensitivity of the neuroprosthesis performance to the choice of PID controller parameters for dorsiflexors. Finally, we applied a first-order linear model for muscle dynamics (Mihelj and Munih, 2004). More accurate modeling of the muscle dynamics could further improve the controller's performance (Rouhani et al., 2016).

In conclusion, we demonstrated the potential of designing PID controllers for closed-loop controlled surface FES applied to ankle muscles to improve standing balance and tested the designed controllers on several individuals and in different experimental paradigms. A PID controller that mimicked the physiological balance control strategy was efficient for this purpose. The balance with closed-loop controlled FES showed improved steady state and transient response compared to the voluntary balance control with disrupted sensory information. The developed methodology can facilitate clinical implementation of a neuroprosthesis for standing balance toward improving the quality of life of neurologically impaired individuals. However, further investigation on clinical implementation of this approach is required.

## Author contributions

HR: Concept/design, Data analysis/interpretation, Drafting article, Approval of article, Statistics; MS: Concept/design, Data collection, Data analysis/interpretation, Drafting article, Approval of article, Statistics; KM: Concept/design, Data analysis/interpretation, Critical revision of article, Approval of article; YL: Data collection, Critical revision of article, Approval of article; MP: Concept/design, Data analysis/interpretation, Critical revision of article, Approval of article. HR and MS equally contributed to preparation of this article.

### Conflict of interest statement

The authors declare that the research was conducted in the absence of any commercial or financial relationships that could be construed as a potential conflict of interest.

## References

[B1] Abbas J. J. Chizeck H. J. (1991). Feedback control of coronal plane hip angle in paraplegic subjects using functional neuromuscular stimulation. IEEE Trans. Biomed. Eng. 38, 687–698. 10.1109/10.83570 1879862

[B2] Ang K. H. Chong G. Li Y. (2005). PID control system analysis, design, and technology. IEEE Trans. Control Syst. Technol. 13, 559–576. 10.1109/TCST.2005.847331

[B3] Aramaki Y. Nozaki D. Masani K. Sato T. Nakazawa K. Yano H. (2001). Reciprocal angular acceleration of the ankle and hip joints during quiet standing in humans. Exp. Brain Res. 136, 463–473. 10.1007/s002210000603 11291727

[B4] Di Giulio I. Baltzopoulos V. Maganaris C. N. Loram I. D. (2013). Human standing: does the control strategy preprogram a rigid knee? J. Appl. Physiol. 114, 1717–1729. 10.1152/japplphysiol.01299.2012 23620493

[B5] Fisher L. E. Miller M. E. Bailey S. N. Davis J. A. Anderson J. S. Rhode L.. (2008). Standing after spinal cord injury with four-contact nerve-cuff electrodes for quadriceps stimulation. IEEE Trans. Neural Syst. Rehabil. Eng. 16, 473–478. 10.1109/TNSRE.2008.2003390 18990650 PMC2936226

[B6] Fitzpatrick R. Rogers D. K. McCloskey D. I. (1994). Stable human standing with lower-limb muscle afferents providing the only sensory input. J. Physiol. 480, 395–403. 10.1113/jphysiol.1994.sp020369 7869254 PMC1155855

[B7] Forrest G. P. Smith T. C. Triolo R. J. Gagnon J. P. DiRisio D. Miller M. E. (2007). Energy cost of the case western reserve standing neuroprosthesis. Arch. Phys. Med. Rehabil. 88, 1074–1076. 10.1016/j.apmr.2007.05.011 17678672

[B8] Gage W. H. Winter D. A. Frank J. S. Adkin A. L. (2004). Kinematic and kinetic validity of the inverted pendulum model in quiet standing. Gait Posture 19, 124–132. 10.1016/S0966-6362(03)00037-7 15013500

[B9] Gollee H. Hunt K. J. Wood D. E. (2004). New results in feedback control of unsupported standing in palaplegia. IEEE Trans. Neural Syst. Rehabil. Eng. 12, 73–80. 10.1109/TNSRE.2003.822765 15068190

[B10] Harkema S. J. Ferreira C. K. van den Brand R. J. Krassioukov A. V. (2008). Improvements in orthostatic instability with stand locomotor training in individuals with spinal cord injury. J. Neurotrauma 25, 1467–1475. 10.1089/neu.2008.0572 19118454 PMC2729458

[B11] Ho C. H. Triolo R. J. Ph D. Elias A. L. Kilgore K. L. Dimarco A. F. (2014). Functional electrical stimulation and spinal cord injury. Phys. Med. Rehabil. Clin. N. Am. 25, 631–ix. 10.1016/j.pmr.2014.05.001 25064792 PMC4519233

[B12] Holderbaum W. Hunt K. J. Gollee H. (2004). Robust discrete H∞ control for unsupported paraplegic standing: experimental results. Eur. J. Control 10, 275–284. 10.3166/ejc.10.275-284

[B13] Hunt K. J. Gollee H. Jaime R. P. (2001). Control of paraplegic ankle joint stiffness using FES while standing. Med. Eng. Phys. 23, 541–555. 10.1016/S1350-4533(01)00089-3 11719077

[B14] Hunt K. J. Munih M. de N Donaldson N. (1997). Feedback control of unsupported standing in paraplegia-part I: optimal control approach. IEEE Trans. Rehabil. Eng. 5, 331–340. 10.1109/86.650287 9422458

[B15] Jaeger R. J. (1986). Design and simulation of closed-loop electrical stimulation orthoses for restoration of quiet standing in paraplegia. J. Biomech. 19, 825–835. 10.1016/0021-9290(86)90133-8 3782165

[B16] Kim J. Y. Mills J. K. Vette A. H. Popovic M. R. (2007). Optimal combination of minimum degrees of freedom to be actuated in the lower limbs to facilitate arm-free paraplegic standing. J. Biomech. Eng. 129, 838–847. 10.1115/1.2800767 18067387

[B17] Kobravi H. R. Erfanian A. (2012). A decentralized adaptive fuzzy robust strategy for control of upright standing posture in paraplegia using functional electrical stimulation. Med. Eng. Phys. 34, 28–37. 10.1016/j.medengphy.2011.06.013 21764350

[B18] Li Y. Ang K. H. Chong G. C. Y. (2006). PID control system analysis and design. IEEE Control Syst. 26, 32–41. 10.1109/MCS.2006.1580152

[B19] Loram I. D. Lakie M. (2001). Balancing of an inverted pendulum: subject sway size is not correlated with ankle impedance. J. Physiol. 532, 879–891. 10.1111/j.1469-7793.2001.0879e.x 11313453 PMC2278569

[B20] Loram I. D. Lakie M. (2002). Direct measurement of human ankle stiffness during quiet standing: the intrinsic mechanical stiffness is insufficient for stability. J. Physiol. 545, 1041–1053. 10.1113/jphysiol.2002.025049 12482906 PMC2290720

[B21] Masani K. (2003). Importance of body sway velocity information in controlling ankle extensor activities during quiet stance. J. Neurophysiol. 90, 3774–3782. 10.1152/jn.00730.2002 12944529

[B22] Masani K. Sayenko D. G. Vette A. H. (2013). What triggers the continuous muscle activity during upright standing? Gait Posture 37, 72–77. 10.1016/j.gaitpost.2012.06.006 22824676

[B23] Masani K. Vette A. H. Kawashima N. Popovic M. R. (2008). Neuromusculoskeletal torque-generation process has a large destabilizing effect on the control mechanism of quiet standing. J. Neurophysiol. 100, 1465–1475. 10.1152/jn.00801.2007 18596181

[B24] Masani K. Vette A. H. Popovic M. R. (2006). Controlling balance during quiet standing: proportional and derivative controller generates preceding motor command to body sway position observed in experiments. Gait Posture 23, 164–172. 10.1016/j.gaitpost.2005.01.006 16399512

[B25] Mihelj M. Munih M. (2004). Unsupported standing with minimized ankle muscle fatigue. IEEE Trans. Biomed. Eng. 51, 1330–1340. 10.1109/TBME.2004.827560 15311817

[B26] Mizrahi J. Levin O. Aviram A. Isakov E. Susak Z. (1997). Muscle fatigue in interrupted stimulation: effect of partial recovery on force and EMG dynamics. J. Electromyogr. Kinesiol. 7, 51–65. 10.1016/S1050-6411(96)00018-1 20719691

[B27] Moore S. P. Rushmer D. S. Windus S. L. Nashner L. M. (1988). Human automatic postural responses: responses to horizontal perturbations of stance in multiple directions. Exp. Brain Res. 73, 648–658. 10.1007/BF00406624 3224674

[B28] Munih M. de N Donaldson N. Hunt K. J. Barr F. M. (1997). Feedback control of unsupported standing in paraplegia–part II: experimental results. IEEE Trans. Rehabil. Eng. 5, 341–352. 10.1109/86.650288 9422459

[B29] Nataraj R. Audu M. L. Kirsch R. F. Triolo R. J. (2010). Comprehensive joint feedback control for standing by functional neuromuscular stimulation-A simulation study. IEEE Trans. Neural Syst. Rehabil. Eng. 18, 646–657. 10.1109/TNSRE.2010.2083693 20923741 PMC3570823

[B30] Nataraj R. Audu M. L. Triolo R. J. (2012). Center of mass acceleration feedback control of functional neuromuscular stimulation for standing in presence of internal postural perturbations. J. Rehabil. Res. Dev. 49, 889–911. 10.1682/JRRD.2011.07.0127 23299260 PMC3573353

[B31] Nataraj R. Audu M. L. Triolo R. J. (2013). Center of mass acceleration feedback control of standing balance by functional neuromuscular stimulation against external postural perturbations. IEEE Trans. Biomed. Eng. 60, 10–19. 10.1109/TBME.2012.2218601 22987499 PMC3578290

[B32] Nataraj R. Audu M. L. Triolo R. J. (2016). Simulating the restoration of standing balance at leaning postures with functional neuromuscular stimulation following spinal cord injury. Med. Biol. Eng. Comput. 54, 163–176. 10.1007/s11517-015-1377-5 26324246 PMC4775462

[B33] Peterka R. J. (2000). Postural control model interpretation of stabilogram diffusion analysis. Biol. Cybern. 82, 335–343. 10.1007/s004220050587 10804065

[B34] Peterka R. J. (2002). Sensorimotor integration in human postural control. J. Neurophysiol. 88, 1097–1118. 10.1152/jn.00605.2001 12205132

[B35] Popović D. B. (2014). Advances in functional electrical stimulation (FES). J. Electromyogr. Kinesiol. 24, 795–802. 10.1016/j.jelekin.2014.09.008 25287528

[B36] Rohde L. M. Bonder B. R. Triolo R. J. (2012). Exploratory study of perceived quality of life with implanted standing neuroprostheses. J. Rehabil. Res. Dev. 49, 265–278. 10.1682/JRRD.2010.08.0156 22773528 PMC4465790

[B37] Rouhani H. Popovic M. R. Same M. Li Y. Q. Masani K. (2016). Identification of ankle plantar-flexors dynamics in response to electrical stimulation. Med. Eng. Phys. 38, 1166–1171. 10.1016/j.medengphy.2016.07.011 27544922

[B38] Same M. (2014). Closed-Loop Control of Ankle Plantarflexors and Dorsiflexors Using an Inverted Pendulum Apparatus. University of Toronto.

[B39] Same M. Rouhani H. Masani K. Popovic M. (2013). Closed-loop control of ankle plantarflexors and dorsiflexors using an inverted pendulum apparatus: a pilot study. J. Autom. Control 21, 31–36. 10.2298/JAC1301031S

[B40] Santos M. J. Kanekar N. Aruin A. S. (2010). The role of anticipatory postural adjustments in compensatory control of posture: 1. Electromyographic analysis. J. Electromyogr. Kinesiol. 20, 388–397. 10.1016/j.jelekin.2009.06.006 19660966

[B41] Tan J. F. (2008). Closed-Loop Control of the Ankle Joint Using Functional Electrical Stimulation. University of Toronto (Toronto, ON).

[B42] Tan J. F. Masani K. Vette A. H. Zariffa J. Robinson M. Lynch C. (2014). Inverted pendulum standing apparatus for investigating closed-loop control of ankle joint muscle contractions during functional electrical stimulation. Int. Sch. Res. Not. 2014:8 10.1155/2014/192097 PMC4897497 27350992

[B43] Tepavac D. Schwirtlich L. (1997). Detection and prediction of FES-induced fatigue. J. Electromyogr. Kinesiol. 7, 39–50. 10.1016/S1050-6411(96)00008-9 20719690

[B44] Triolo R. J. Bailey S. N. Miller M. E. Loretta M. Anderson J. S. Davis J. A. Jr. (2012). Longitudinal performance of a surgically implanted neuroprosthesis for lower extremity exercise, standing, and transfers after spinal cord injury. Arch. Phys. Med. Rehabil. 93, 896–904. 10.1016/j.apmr.2012.01.001 22541312 PMC4111081

[B45] Vette A. H. Masani K. Kim J. Popovic M. R. (2009). Closed-loop control of functional electrical stimulation-assisted arm-free standing in individuals with spinal cord injury: a feasibility study. Neuromodulation 12, 22–32. 10.1111/j.1525-1403.2009.00184.x 22151219

[B46] Vette A. H. Masani K. Nakazawa K. Popovic M. R. (2010). Neural-mechanical feedback control scheme fluctuation during quiet stance. IEEE Trans. Neural Syst. Rehabil. Eng. 18, 86–95. 10.1109/TNSRE.2009.2037891 20071280

[B47] Vette A. H. Masani K. Popovic M. R. (2007). Implementation of a physiologically identified PD feedback controller for regulating the active ankle torque during quiet stance. IEEE Trans. Neural Syst. Rehabil. Eng. 15, 235–243. 10.1109/TNSRE.2007.897016 17601193

[B48] Welch T. D. J. Ting L. H. (2009). A feedback model reproduces muscle activity during human postural responses to support-surface translations. J. Neurophysiol. 1032–1038. 10.1152/jn.01110.2007 18094102

[B49] Winter D. A. (1995). Human blance and posture control during standing and walking. Gait Posture 3, 193–214. 10.1016/0966-6362(96)82849-9

